# Preventing antenatal stillbirths: An innovative approach for primary health care

**DOI:** 10.4102/safp.v64i1.5487

**Published:** 2022-08-25

**Authors:** Tsakane M. Hlongwane, Tanita Botha, Bongani S. Nkosi, Robert C. Pattinson

**Affiliations:** 1 Department of Obstetrics and Gynaecology, Faculty of Health Sciences, University of Pretoria, Pretoria, South Africa; 2 Research Centre for Maternal, Fetal, Newborn and Child Health Care Strategies, Faculty of Health Sciences, University of Pretoria, Pretoria, South Africa; 3 Maternal and Infant Health Care Strategies Research Unit, South African Medical Research Council, Faculty of Health Sciences, University of Pretoria, Pretoria, South Africa; 4 Department of Statistics, Faculty of Natural Sciences and Agricultural Sciences, University of Pretoria, Pretoria, South Africa

**Keywords:** antenatal care, pregnancy, primary healthcare clinics, stillbirths, Doppler, umbilical artery blood flow, foetal growth restriction

## Abstract

**Background:**

In South Africa (SA), approximately 16 000 stillbirths occur annually. Most are classified as unexplained and occur in district hospitals. Many of these deaths may be caused by undetected foetal growth restriction. Continuous wave Doppler ultrasound of the umbilical artery (CWDU-UmA) is a simple method for assessing placental function. This screening method may detect the foetus at risk of dying and growth-restricted foetuses, allowing for appropriate management.

**Methods:**

A cohort study was conducted across South Africa. Pregnant women attending primary health care clinics at 28–34 weeks gestation were screened using CWDU-UmA. Women not screened at those antenatal clinics served as control group 1. Control group 2 consisted of the subset of control group 1 with women detected with antenatal complications excluded. Women with foetuses identified with an abnormal CWDU-UmA test were referred and managed according to a standardised protocol. A comparison between the study and control groups was performed.

**Results:**

The study group consisted of 6536 pregnancies, and there were 66 stillbirths (stillbirth rate [SBR]: 10.1/1000 births). In control group 1, there were 193 stillbirths in 10 832 women (SBR: 17.8/1000 births), and in control group 2, 152 stillbirths in 9811 women (SBR: 15.5/1000 births) (risk ratio: 0.57, 95% confidence intervals: 0.29–0.85 and 0.65, 0.36–0.94, respectively).

**Conclusion:**

Screening a low-risk pregnant population identified the low-risk mother with a high-risk foetus, and acting on the information as described was associated with a significant reduction (35% – 43%) in stillbirths. This demonstrates a step-change reduction in stillbirths and warrants screening in SA.

## Introduction

Stillbirths are mostly hidden. Few clinicians, except for the midwife and doctor directly involved, see the stillbirth, and the woman (mostly) does not discuss the event with others outside of the immediate family. Stillbirth leads to great anguish for the health care providers and the family.^1,2^

Stillbirths are a global public health challenge and the majority occur in low- and middle-income countries (LMICs).^1,2^ Approximately 2.0 million stillbirths occur worldwide each year.^2^ Approximately 55% of stillbirths occur in sub-Saharan Africa.^2,3^ South Africa (SA) is classified as an upper middle-income country and is ranked 50th of 54 similar countries, and within Africa, SA is ranked 6th of the seven upper middle-income countries.^2^

District hospitals and community health centres reported 2.5 stillbirths for every neonatal death, and 43% of the approximately 16 000 stillbirths per year (foetal weight of 1000 g or more) occur in SA.^4^ Perinatal deaths include both stillbirths (defined as an infant born with no signs of life at 1 min and 5 min, Apgar zero) and a neonatal death (defined as a death of a neonate in the first 28 days of life). The most common category of perinatal deaths is unexplained stillbirth, which accounts for approximately a quarter of perinatal deaths (30% in district hospitals and community health centres) and for 36% of stillbirths (52% of these stillbirths were in the district hospitals and community health centres).^3,4,5^ The majority of the mothers and foetuses were regarded as healthy at the time of the foetal demise and had not been referred for more specialised care.^2,3,4,5^ Foetal growth restriction (FGR) is a major factor associated with stillbirth.^5,6,7,8,9^

Small for gestational age (SGA) foetuses (defined as babies born < 10th centile for gestational age and used as a surrogate of FGR) not detected during the antepartum period had a fourfold increased risk of adverse foetal complications compared to SGA foetuses detected before delivery.^6,7,8,9,10,11^ Foetal growth restriction increases the risk of adverse outcomes by eightfold and is associated with perinatal morbidity and mortality and adulthood diseases.^9,12,13^ Three-quarters of growth-restricted infants are not recognised before delivery,^6,7,8,9,10,11,12,13^ and in low-risk pregnancies with a lower threshold of suspicion, the detection rate is even lower (15% – 20%).^3,4,5,6,7,8,9,10,11,12,13^

The tools required to detect FGR in LMICs and especially at the level of primary care, where most women with low-risk pregnancies attend, perform extremely poorly in detecting FGR, potentially explaining the high unexplained stillbirth rate. Palpation and symphysis-fundal height measurement are commonly used in LMICs to detect FGR, despite the limited evidence to support this as an effective method to detect growth restriction and show improved maternal or neonatal outcomes.^14^ Symphysis-fundus measurements have a poor ability to detect growth-restricted babies, and routine foetal movements counting has also been shown to be ineffective.^14,15^ Routine imaging ultrasound^16^ in LMICs, surprisingly, was also shown to have no effect on perinatal or maternal death or on antenatal attendance.^14,15,16^

Better detection of FGR babies is desperately needed in SA and other LMICs, especially at the primary level of care. Doppler ultrasound of the umbilical artery measures the arterial blood velocity through the placenta.^17^ Reduced blood flow because of increased resistance downstream in the placenta leads to ineffective transfer of nutrients and oxygen and correlates very well with placental insufficiency, which is one of the major causes of FGR.^17,18^ This reduced blood flow is detected by the Doppler ultrasound and measured as the resistance index (RI = peak systolic-end diastolic/peak systolic, decreases with increasing gestational age): the higher the RI, the more compromised the blood flow.^17,18,19^ A rise in RI is associated with FGR, and once absent end-diastolic flow (AEDF) is detected, there is end-stage placental disease; this is associated with adverse perinatal outcomes.^17,18^ When the placenta is damaged to the extent that no blood flow can be detected during diastole of the foetal heart, the placenta is close to failure and the foetus is at high risk of intra-uterine demise. This phenomenon is called AEDF.^18,19^ Clinical care informed by the result of the Doppler ultrasound of the umbilical artery has been shown to reduce perinatal deaths by more than one-third in high-risk pregnancies.^20^ Another Cochrane systematic review found there was insufficient evidence to support its use in low-risk pregnancies.^21^ However, this recommendation was based on study populations from high-income countries. Screening for AEDF, or a clearly defined abnormally high RI, could potentially reduce stillbirths if the prevalence of AEDF and abnormal RI was high enough in a pregnant population to warrant screening. These circumstances might be found in LMICs with high stillbirth numbers.

The Umbiflow^TM^ device is a low-cost mobile CWDU device developed by the Council for Scientific and Industrial Research (CSIR) and SAMRC in South Africa.^22^ It measures the RI in the umbilical artery and plots it against the estimated gestational age to identify the foetus at risk for FGR.^22,23^ The accuracy of Umbiflow^TM^ in measuring the RI in the foetal umbilical artery continuous wave Doppler ultrasound of the umbilical artery (CWDU-UmA) has been proven to be comparable to the commercial standard unit ‘gold standard’.^23^ The device has significant advantages in that it can be used by primary health care providers and non-specialist healthcare providers, requires only a week of training, is portable and is less expensive and less technical than imaging ultrasound; the data are recorded on the device, which allows for quality control at a later date, and it can run on battery power.^23,24^ These factors make it a good choice for a screening tool if it is effective in detecting FGR. A previous study performed in Mamelodi township using Umbiflow^TM^ found that the use of Umbiflow^TM^ reduced the stillbirth rate in women classified as having low-risk pregnancies by 43%.^24^

Building on the previous study, we investigated whether screening a low-risk pregnant population using Umbiflow^TM^ in primary healthcare clinics throughout SA, together with a standard referral protocol for foetuses with abnormal RI, would result in a reduction in the stillbirth rate.

## Methods

### Study design

A cohort of low-risk pregnant women attending primary health care antenatal care eligible for screening using the CWDU-UmA (Umbiflow^TM^ device) were screened. The population of low-risk women were defined as women attending non-specialist primary antenatal care clinics who have been classified as ‘low risk’ at the time of recruitment and screening according to local clinical guidelines. This is based on the South African basic antenatal care plus guideline grounded on the World Health Organization (WHO) guideline.^25,26^

The study started with recruitment and screening in September 2017. There were nine study sites across eight provinces in SA. The different sites started at different times to allow for adequate training and quality control at all of the nine sites.^27^

### Study participants

Women from a specific geographic area attending the primary health care clinic were considered for screening. The inclusion criteria were as follows: all candidates must be women with a singleton pregnancy, aged 18 years or more and classified as low risk between 28 and 34 weeks gestation (if the gestational age [GA] was unknown, a symphysis-fundal height of 26 cm or above was used); all candidates were also required to provide written consent.^27^ Women were screened with CWDU-UmA on specific days of the week, and those not attending the clinics on those days served as the control group. A comparison between those who were screened using CWDU-UmA (study group) and those not screened (control group) was performed. Control group 1 represents all women aged 18 years or more who attended the antenatal clinics, had a singleton pregnancy, delivered a neonate of 1000 g or more from the clinic and did not receive CWDU-UmA screening; control group 2 represents the same population, but excludes those who subsequently developed antenatal complications (hypertensive disorders of pregnancy, prolonged rupture of membranes, diabetes mellitus, antepartum haemorrhage and sepsis). This was to ensure that the screened population was as low-risk a population as possible.

### Study setting

The study sites included nine diverse catchment areas linked to regional or tertiary hospitals. This included rural, peri-urban and urban sites across eight provinces in SA. The sites had a healthcare worker screening eligible women on specific days of the week, and there was an established communication and referral route to refer women with abnormal RI.^27^

### Measurement

The CWDU-UmA screening was classified as either normal RI or abnormal RI, depending on the Doppler value in relation to GA and RI. This was automatically plotted on a graphic representation using the 75th centile as a cut-off.^19^ Screened women with RI findings below the 75th centile for their GA were considered as having a normal RI result, and they continued their routine antenatal care at their local primary health care clinics.^27^ The Mamelodi study found that approximately 10% of a low-risk population with abnormal RI was identified by using the 75th centile cut-off.^24^ Those with RI findings above the 75th centile for their GA were considered to have abnormal RI and were referred to a high-risk clinic at their local referral hospitals for further review and management.^24,27^

**FIGURE 1:**
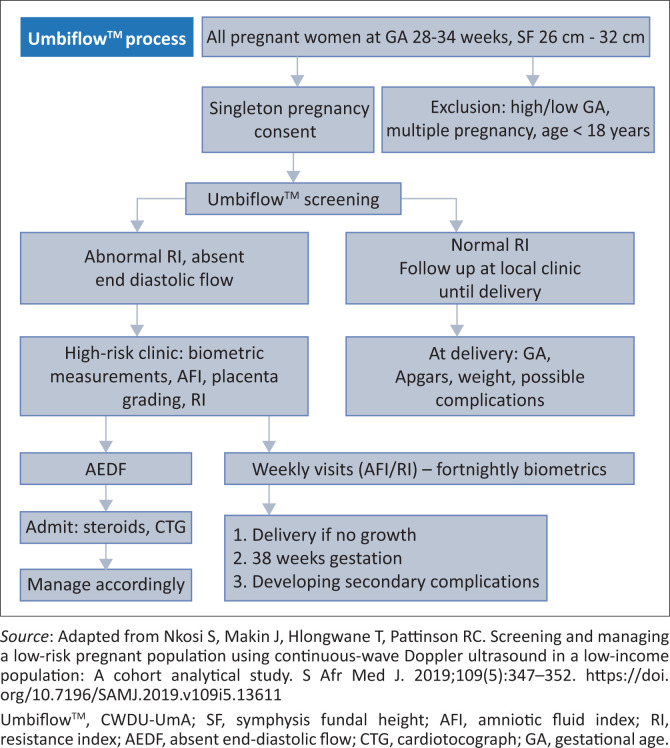
Flow diagram of screened population management.

Women with abnormal RIs were followed up weekly or fortnightly at the high-risk clinic and received a Doppler ultrasound at each visit and a foetal growth scan every two weeks. All were managed according to standard protocol. Figure 1 illustrates the management protocol for women with an abnormal RI Umbiflow^TM^ result. The stillbirth rates in the screened population (study group) were compared with those of control groups 1 and 2.

### Data collection

Data collection of the screened women and delivery outcomes started in September 2017 and stopped in February 2020, allowing time for the pregnant women to deliver by the end of February 2020. The CWDU-UmA screening outcome was recorded. Maternal clinical information was collected at the time of enrolment in the study. The outcome of all women screened (study group) or not screened (control groups) was recorded. Outcome data were obtained from the electronic birth register at the various delivery sites. Small for gestational age was defined as a birth weight for gestational age < 10th centile, according to the WHO foetal growth chart.^28^

### Statistical analysis

The maternal demographics are reported as frequencies, means and standard deviations. Categorical characteristics were investigated using chi-square tests to express differences between the screened and not screened groups and the two-proportion *z*-tests for cases where only certain categories were compared. The WHO multinational foetal growth charts were used for categorising birth weight according to centile and corrected for GA and neonatal sex at delivery.^28^ All tests were performed at a 5% level of significance. A *p*-value of < 0.05 was considered as statistically significant. The relative risk was calculated using the incidence proportions of total stillbirth rate and perinatal mortality rate between the screened and not screened groups. All statistical analyses were done with R Core Team.^27^

### Ethical considerations

All procedures performed in studies involving human participants were in accordance with the ethical standards of the institutional and/or national research committee and with the 1964 Helsinki Declaration and its later amendments or comparable ethical standards. All participants provided written informed consent. Participation was voluntary and participants were informed about their rights to withdraw from the study at any stage. Ethical approval was obtained from the University of Pretoria’s Faculty of Health Sciences (clearance no. 473/2014).

## Results

During the study period, there were 20 330 women who attended antenatal care at the designated primary health care clinics and delivered at the clinic or hospital. After excluding women below the age of 18 years, deliveries below 28 weeks’ gestation (or women who gave birth to newborns weighing less than 1000 g if GA was unknown) and women with no maternal and neonatal clinical notes available and multiple pregnancies, 17 368 pregnancies were analysed.

**FIGURE 2:**
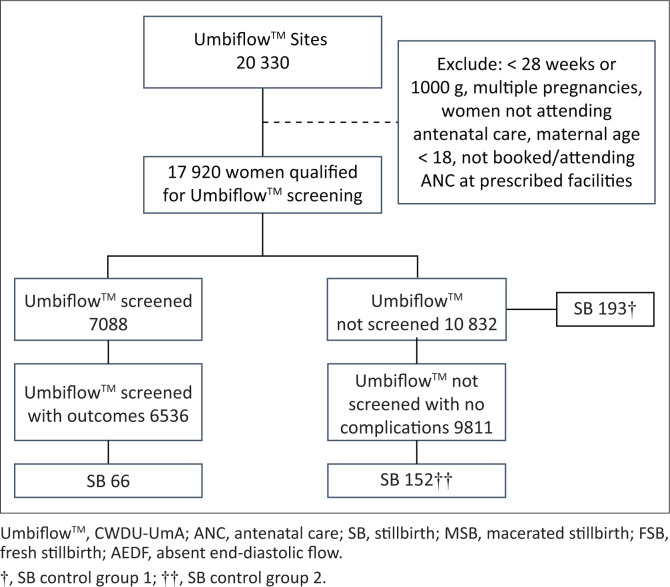
Flow chart of women included in the study.

The screened study group consisted of 7088 women who had CWDU-UmA screening, of which 6536 (92.2%) women had pregnancy outcomes that were analysed. Control group 1 had 10 832 women, and control group 2 had 9811 women. Figure 2 gives a breakdown of the women included in the study.

The incidence of abnormal RI in the screened group was 13.0%, and 1.2% of the screened population had AEDF. Details are documented elsewhere.^27^

Table 1 gives a comparison between the study group and the two control groups. There was no difference in the age of study group and control groups or in HIV status; however, the study group had significantly more nulliparous women. The control groups had more low-birthweight babies than the study group, but the study group had more SGA babies and more admissions to neonatal nursery. There was no difference in the mode of delivery between the study group and control group 1 (caesarean section: 29.5% vs 28.5%, *p* = 0.155), but there were more caesarean section deliveries in the study group than in control group 2 (29.5% vs 26.4%, *p* = 0.0001). A *p*-value of < 0.05 was considered as statistically significant.

**TABLE 1a:** Comparison of the continuous wave Doppler ultrasound of the umbilical artery screened group and control groups 1 and 2.

Indicator	Total screened (*n* = 6536)				Not screened (control 1) (*n* = 10 832)				Screened versus control 1 - *p*-value	Not screened, no ANC complications (control 2) (*n* = 9811)				Screened versus control 2 - *p*-value
	*n*	%	Mean	s.d.	*n*	%	Mean	s.d.		*n*	%	Mean	s.d.	
**Demographic information on the CWDU-UmA screened population**														
Age (years)														
18–19	465	7.1	-	-	785	7.2	-	-	0.3421	690	7.0	-	-	0.2770
20–34	4993	76.4	-	-	8351	77.1	-	-	-	7592	77.4	-	-	-
35+	1078	16.5	-	-	1696	15.7	-	-	-	1529	15.6	-	-	-
Parity														
0–0	2179	33.3	-	-	2886	26.6	-	-	< 0.0001*	2537	25.9	-	-	< 0.0001*
1–4	4288	65.6	-	-	7762	71.7	-	-	-	7108	72.4	-	-	-
5+	69	1.1	-	-	184	1.7	-	-	-	166	1.7	-	-	-
HIV														
positive	2002	30.6	-	-	3305	30.5	-	-	0.8825	2973	30.3	-	-	0.6682
negative	4534	69.4	-	-	7527	69.5	-	-	-	6838	69.7	-	-	-
**Outcomes information of the CWDU-UmA screened population**														
Birthweight (g) categories at delivery														
1000 g – 1499 g	31	0.5	-	-	235	2.2	-	-	< 0.0001*	174	1.8	-	-	< 0.0001*
1500 g – 1999 g	124	1.9	-	-	357	3.3	-	-	-	276	2.8	-	-	-
2000 g – 2499 g	575	8.8	-	-	854	7.9	-	-	-	752	7.7	-	-	-
> 2500 g	5806	88.8	-	-	9386	86.7	-	-	-	8609	87.7	-	-	-
Birth weight	-	-	3074	517	-	-	3050	552	-	-	-	3059	536	-
GA at birth (weeks)	-	-	39	1.8	-	-	38	2.3	-	-	-	38	2.2	-
LBW < 2500	730	11.2	-	-	1446	13.3	-	-	< 0.0001*	1202	12.3	-	-	0.0379*
SGA† (10th centile)	1580	24.2	-	-	2235	20.6	-	-	< 0.0001*	2002	20.4	-	-	< 0.0001*
Admission nursery	420	6.4	-	-	363	3.4	-	-	< 0.0001*	226	2.3	-	-	< 0.0001*
Delivery mode														
Caesarean section	1930	29.5	-	-	3088	28.5	-	-	0.1555	2591	26.4	-	-	< 0.0001*
Vaginal delivery	4606	70.5	-	-	7744	71.5	-	-	-	7220	73.6	-	-	-

Note: Data are n/N (%).

GA, gestational age; LBW, low birthweight; CS, caesarean section; NVD, normal vaginal delivery; RR, risk ratio; SB, stillbirth; SBR, Stillbirth rate; CWDU-UmA, continuous wave Doppler ultrasound of the umbilical artery; ANC, antenatal care; s.d., standard deviation.

* , Signifies statistical significance at 95% confidence interval.

† , SGA determined using the World Health Organization growth charts.

**TABLE 1b:** Comparison of the continuous wave Doppler ultrasound of the umbilical artery screened group and control groups 1 and 2.

Indicator	Total screened (*n* = 6536)		Not screened (control 1) (*n* = 10 832)				Not screened (control 2) (*n* = 9811)			
	*n*	Per 1000	*n*	Per 1000	RR	95% CI	*n*	Per 1000	RR	95% CI
**Impact on the CWDU-UmA screened population**										
Impact										
Number (SBR/1000)	66	10.1	193	17.8	0.57	0.29–0.85	152	15.5	0.65	0.36–0.94

Note: Data are n/N (%).

RR, risk ratio; CI, confidence interval; SBR, stillbirth rate; CWDU-UmA, continuous wave Doppler ultrasound of the umbilical artery.

There were 66 stillbirths in the screened group (53 normal RI and 13 abnormal RI), 193 in the control group 1 and 152 in control group 2. The stillbirth rate was significantly lower in the CWDU-UmA screened study group compared with both control groups (study group vs control 1:10.1/1000 births vs 17.8/1000 births, risk ratio [RR]: 0.57, 95% confidence interval [CI]: 0.29–0.85; study group vs control 2:10.1/1000 vs 15.5/1000, RR: 0.65, 95% CI: 0.36–0.94).

Table 2 describes all the primary causes of stillbirths in the CWDU-UmA screened study group. There were nine neonatal deaths in the screened group (neonatal death rate 1.4/1000 live births); two were because of intrauterine growth restriction, two because of congenital abnormalities (cardiac and multiple abnormalities), two because of hypertensive disorders in pregnancy, and one was because of intrapartum asphyxia. In a further two cases, no obstetric cause could be identified; one neonate died at home, thought to be because of aspiration following breastfeeding, and the other died unexpectedly in the postnatal ward and was classified as sudden infant death syndrome.

**TABLE 2a:** Primary causes of stillbirths and neonatal deaths for the continuous wave Doppler ultrasound of the umbilical artery screened study group.

Primary causes of stillbirths in the screened group	CWDU-UmA screened SB (*n* = 66)
Proteinuric hypertension	3
Eclampsia	0
Unexplained intrauterine death – macerated	30
Unexplained intrauterine death – fresh	3
Abruptio placentae	1
Labour-related intrapartum asphyxia	16
Cord around the neck	1
Meconium aspiration	1
Cord prolapse	1
Traumatic breech delivery	1
Amniotic fluid infection	1
Foetal chromosomal abnormality	1
Abnormality of multiple systems	3
Idiopathic intrauterine growth restriction	3
Postmaturity	1

N, number of cases; SB, stillbirth; CWDU-UmA, continuous wave Doppler ultrasound of the umbilical artery.

**TABLE 2b:** Primary causes of stillbirths and neonatal deaths for the continuous wave Doppler ultrasound of the umbilical artery screened study group.

Primary causes of neonatal deaths in screened group	CWDU-UmA screened NND (*n* = 9)
Proteinuric hypertension	2
Labour-related intrapartum asphyxia	1
Abnormality of multiple systems	1
Cardiovascular system abnormality	1
Idiopathic intrauterine growth restriction	2
No obstetric cause/not applicable/unknown	2

N, number of cases; NND, neonatal death; CWDU-UmA, continuous wave Doppler ultrasound of the umbilical artery.

Unfortunately, we have been unable to reliably trace all the neonatal outcomes of the women that attended the same primary health care clinics as the study group. Our electronic birth register records all stillbirths, but it does not record all the neonatal deaths. The Perinatal Problem Identification Programme records all the neonatal deaths for the maternity units, but it is not granular enough to identify the clinics that the women attended. The overall neonatal deaths for the nine catchment areas were 658 neonates from 69 301 live births for women aged 18 years or older, who attended antenatal care, had a singleton pregnancy and delivered an alive baby of 1000 g or more (excluding the neonates of the study group). This gives a neonatal death rate of 9.5/1000 live births. The major neonatal causes included intrapartum asphyxia (29.9%), spontaneous preterm labour (29.3%), foetal anomalies (10.5%), HDP (10.3%), antepartum haemorrhage (4.9%) and congenital infections (3.8%).

## Discussion

Screening a low-risk pregnant population with CWDU-UmA at primary care clinics and referral of foetuses with abnormal RIs to the next level of care resulted in a significant (43%) reduction in the stillbirth rate (RR: 0.57, 95% CI: 0.29–0.85); even with the unscreened population who developed antenatal complications excluded, there was a significant (35%) reduction in the stillbirth rate (control group 2, RR: 0.65, 95% CI: 0.36–0.94). There was no increase in the neonatal death rate that was discernible. This reduction in the stillbirth rate was achieved with only a slight increase in resources, namely increased use of the neonatal nursery (6.4% vs 3.4% vs 2.3, *p =* 0.0001) and increased caesarean section rate (29.5% vs 28.5%, *p* = 0.155 in control group 1 and 29.5% vs 26.4%, *p* = 0.0001 in control group 2). This finding is similar to the Mamelodi study which showed a reduction in stillbirths, an increase in preterm deliveries without increasing neonatal mortality.^24^

The incidence of AEDF at 1.2% in the low-risk pregnant population and setting the abnormal RI at approximately 10% of the population meant that for every 100 women screened, 10 women were referred, and of these 10 women, one foetus had end-stage placental disease.^27^ This incidence warrants screening at a primary care level, given that the natural history of AEDF is a stillbirth.^24,27^

The largest category of stillbirths delivering at district hospitals and community health centres is unexplained stillbirth, and the majority of the mothers are clinically healthy, but the majority of these stillbirths had undetected FGR or were SGA.^3,4,5,6,7,8,9,10,11,12,13^ It is well documented that growth failure is associated with adverse perinatal outcomes and disease later in adulthood.^6,7,8,9,10,11^ Antenatal care of apparently healthy women occurs mainly at primary health care clinics, and to reduce the antenatal stillbirth rate, the clinicians at the primary health care clinics need to identify the high-risk foetus (mostly growth-restricted) in the healthy woman. Current methods of detecting FGR are ineffective.^14,15,16^ We now know that CWDU-UmA is a feasible tool to detect FGR, and its use in screening at primary health care clinics is associated with a step-change reduction in stillbirths, thus potentially solving this problem.

There was no difference in age or HIV status between the study group and control groups, but the screened group had more primigravid women. Primigravid women are more likely to attend antenatal care more frequently,^29^ and as such they were more likely to have attended on the days of the Umbiflow^TM^ screening. However, there were more low-birthweight (LBW) babies in the control groups (13.3% and 12.3% vs 11.2% in the study group), but the study group had more SGA babies (24.2% vs 20.6% and 20.2%). This imbalance between the study group and the control groups might have been avoided if we had screened every day of the week and if a randomised trial had been conducted.

Other limitations of this study include that CWDU-UmA screening was only performed in women aged 18 years or more, and women were screened on specific days of the week; those not attending on those days did not get a CWDU-UmA screening. We chose not to randomise women so that a larger sample of women could be recruited in a shorter period of time for budgetary reasons. There was some loss to follow-up, but unknown outcomes were only 7.8%, and this is particularly good for low-income and middle-income settings where women are considerably more mobile and relocate frequently. Another limitation of the study was its inability to trace the neonatal outcomes of all the women that attended the same clinics as the screened study group. We did not have a neonatal follow-up system that was granular enough to record neonatal deaths at clinic level, and the contact details in the birth register were insufficient to trace all the neonates from the specific clinics. However, the neonatal outcomes of all the neonates whose mothers were screened were available, as we had complete contact details of the women. This data indicates that there was not a shift to an increased neonatal death rate in the screened group, despite significantly reducing the stillbirths.

A strength of the study is in having two control groups; control group 2 excluded women who developed antenatal complications. We did not remove women from the study group who developed complications, and if anything, they were a slightly higher-risk group than control group 2. This demonstrated that the screened population compared with as low-risk a population as possible with similar conditions and still had a 35% (RR: 0.65, 95% CI: 0.36–0.94) reduction in stillbirths. This reinforces the value of screening the umbilical artery for an abnormal RI in low-risk populations. Another strength of the study was the use of nine different geographical sites and the large number of women involved.

Of the 66 stillbirths in the study group, 30 were still unexplained. Only one screening test was performed around the 30-week visit. It is possible that a subsequent screening at a later time (e.g. 36 weeks) in those women whose foetuses had a normal RI might detect more foetuses at risk. Further research is needed to answer this question. Alternatively, these foetuses might have died because of undiagnosed congenital infection, as described by Madhi et al.^30^

This study demonstrates that screening for FGR using CWDU-UmA was feasible at primary health care clinics and highly effective in preventing stillbirths. As mentioned previously, this screening system is ideal for a primary health care setting. It is recommended that CWDU-UmA should be used to screen pregnant populations for abnormal RIs of the umbilical arteries. Research should concentrate on the timing of screening and methods to implement the CWDU-UmA screening in primary health care clinics in districts throughout SA. Special attention should be paid to the resources that would be required at the referral hospital, namely increased maternity and neonatal high care and intensive care resources.

## Conclusion

Continuous wave Doppler ultrasound of the umbilical artery offers the solution to provide a simple and accurate method of detecting FGR in pregnant women and preventing stillbirths. The high prevalence of AEDF found in SA warrants population screening.
